# Refining the Phenotypic Spectrum of *KMT5B*-Associated Developmental Delay

**DOI:** 10.3389/fped.2022.844845

**Published:** 2022-03-30

**Authors:** Aviva Eliyahu, Ortal Barel, Lior Greenbaum, Gal Zaks Hoffer, Yael Goldberg, Annick Raas-Rothschild, Amihood Singer, Ifat Bar-Joseph, Vered Kunik, Elisheva Javasky, Orna Staretz-Chacham, Naomi Pode-Shakked, Lily Bazak, Noa Ruhrman-Shahar, Elon Pras, Moshe Frydman, Mordechai Shohat, Ben Pode-Shakked

**Affiliations:** ^1^The Danek Gertner Institute of Human Genetics, Sheba Medical Center, Ramat Gan, Israel; ^2^Sackler Faculty of Medicine, Tel-Aviv University, Tel-Aviv, Israel; ^3^The Genomic Unit, Sheba Cancer Research Center, Sheba Medical Center, Ramat Gan, Israel; ^4^The Wohl Institute for Translational Medicine and Cancer Research Center, Sheba Medical Center, Ramat Gan, Israel; ^5^The Joseph Sagol Neusroscience Center, Sheba Medical Center, Ramat Gan, Israel; ^6^The Raphael Recanati Genetics Institute, Rabin Medical Center - Beilinson Hospital, Petah Tikva, Israel; ^7^The Institute for Rare Diseases, Edmond and Lily Safra Children's Hospital, Sheba Medical Center, Ramat Gan, Israel; ^8^Department of Community Genetics, Public Health Services, Ministry of Health, Jerusalem, Israel; ^9^Bioinformatics Consulting, Maas, Israel; ^10^Metabolic Clinic, Soroka Medical Center, Be'er Sheva, Israel; ^11^Faculty of Health Sciences, Ben-Gurion University, Be'er Sheva, Israel; ^12^Department of Pediatrics, Edmond and Lily Safra Children's Hospital, Sheba Medical Center, Ramat Gan, Israel; ^13^The Talpiot Medical Leadership Program, Sheba Medical Center, Ramat Gan, Israel; ^14^Mina and Everard Goodman Faculty of Life Science, Bar Ilan University, Ramat Gan, Israel; ^15^Felsenstein Medical Research Center, Rabin Medical Center, Petah Tikva, Israel

**Keywords:** *KMT5B*, intellectual disability, developmental delay, *de novo*, macrocephaly

## Abstract

The role of lysine methyltransferases (KMTs) and demethylases (KDMs) in the regulation of chromatin modification is well-established. Recently, deleterious heterozygous variants in *KMT5B* were implicated in individuals with intellectual disability (ID) and/or autism spectrum disorder. We describe three unrelated patients with global developmental delay (GDD) or ID, macrocephaly and additional features. Using whole exome sequencing, each of the probands was found to harbor a distinct *de novo* heterozygous disease-causing variant in *KMT5B*: c.541C > G (p.His181Asp); c.833A > T (p.Asn278Ile); or c.391_394delAAAG (p.Lys131GlufsTer6). We discuss herein their clinical presentations, and compare them to those of previously reported patients. Furthermore, using a three-dimensional computational model of the *KMT5B* protein, we demonstrate the predicted structural effects of the two missense variants. Our findings support the role of *de novo* missense and nonsense variants in *KMT5B*-associated GDD/ID, and suggest that this gene should be considered in the differential diagnosis of neurodevelopmental disorders accompanied by macrocephaly and/or overgrowth.

## Introduction

Methyltransferases (KMTs) and demethylases (KDMs) play an important role in the regulation of chromatin modification (1, 2). Pathogenic variants in several of these genes have been reported in autosomal-dominant or X-linked neurodevelopmental disorders. Most recently, heterozygous disease-causing variants in the Lysine Methyltransferase 5B (*KMT5B*) gene, have been described in individuals with neurodevelopmental delay, with or without seizures and/or autism spectrum disorder (ASD) (MIM #617788).

Initially, *KMT5B* (also termed SUV420H1) was suggested as a candidate gene associated with ASD, using computational modeling (3). Only in 2017, in a cohort of ~11,700 patients with neurodevelopmental delay, Stessman et al. found deleterious heterozygous variants in *KMT5B* in seven probands (4). In addition to intellectual disability (ID) (7/7), other features included ASD (5/6), febrile seizures (3/5) and attention deficit (3/7). Of these, three patients had previously been reported as part of a cohort of 2,500 ASD cases (5). Since then, several additional individuals with varying degrees of ID harboring *de novo* heterozygous *KMT5B* variants were reported (6, 7).

Functionally, the role of *KMT5B* in habituation learning was demonstrated in a Drosophila model (4). Most recently, two mouse models for *KMT5B* were reported. Using a comprehensive neurodevelopmental test battery, Wickramasekara et al. showed differences with regard to anxiety, depression, fear and extinction learning in mice haploinsufficient for *KMT5B* compared to controls, as well as differential effects by sex (8, 9). Wang et al. knocked down *Kmt5b* in the mouse prefrontal cortex and demonstrated social deficits, disruption of glutamatergic synaptic transmission as well as upregulation of genes involved in cellular stress response and ubiquitin-dependent protein degradation (10).

We describe herein clinical and molecular features of three unrelated patients with *de novo* disease-causing variants in *Kmt5b*, demonstrate the missense variants' predicted effects in a three dimensional (3D) computational model of the protein, and compare the patients' phenotypic features to those of previously-described patients.

## Materials and Methods

Following parental written informed consent, blood samples from the probands and their parents were obtained for trio Whole exome sequencing (WES). The study was approved by the Institutional Review Board of Sheba Medical Center. WES analysis was performed at the Genomic Unit of the Sheba Cancer Research Center (Probands A,B). These two individuals were included in our previous publication, describing our experience with 280 individuals with neurodevelopmental disorders or multiple congenital anomalies (11). However, the current study describes their clinical phenotype in detail. Proband C was not reported previously, and WES was performed at the Raphael Recanati Genetic Institute at Beilinson Medical Center, following IRB approval.

### Whole Exome Sequencing

Trio-based Whole Exome Sequencing (WES) was performed on genomic DNA at the Genomic Unit, Sheba Medical Center (for families A and B), or at the Raphael Recanati Genetics Institute (for family C), using the Twist Human Core Exome Plus Kit (Twist Bioscience, San Francisco, CA, USA). Detailed methods and technologies used for sequencing, variant calling and interpretation are described in the Supplementary Material.

### 3D Modeling of Wild-Type *KMT5B* and H181D and N278I Variants

The 3D structural models of wild-type (WT) and mutated *KMT5B* structures were constructed using the SWISS-MODEL protein structure homology-modeling server (12), using the PDB structure 5cpr as the template for structure prediction. The same 3D modeling algorithm was used to construct WT and mutated structures to avoid differences that may arise due to the modeling process.

### Structural Analysis

Discovery Studio® Visualizer (version 16.1.0.15350, BIOVIA, Dassault Systèmes Discovery Studio Modeling Environment, Release 4) was used to calculate intra-structure hydrogen bonds and the distances between atoms.

## Results

### Clinical Characteristics

Proband A presented at 12 months of age due to global developmental delay (GDD). The first child born to non-consanguineous healthy parents of mixed Jewish descent, he was born after an uneventful pregnancy and delivery at term (birth weight, 3,212 g). At 3 months of age, neurological and ophthalmological evaluation was pursued due to lack of eye contact. Delay in acquisition of developmental milestones was noted: he raised his head at 5.5 months, rolled over at 7 months and began crawling at 9 months of age. He showed profound GDD and macrocephaly. Parents noted episodes of unexplained laughter. At presentation at the age of 12 months, he was planned to undergo surgical repair of lateral strabismus. Upon physical examination, a broad forehead, strabismus, short neck and mild hypertelorism were noted. Weight was 10 kg (40th percentile) and head circumference was 49.2 cm (>97th percentile). Electroencephalogram (EEG), brain ultrasound and magnetic resonance imaging (MRI) were all intact.

Proband B presented at 2.5 years of age, due to GDD and relative macrocephaly. He was the first child of healthy, non-consanguineous Ashkenazi-Jewish parents, and born following a spontaneous, uneventful pregnancy, via post-term delivery (birth weight, 3,800 g). During infancy, brain ultrasound was performed due to large head circumference, and was normal. At 12 months he began passing loose stools, and was diagnosed with lactose intolerance. Ophthalmological evaluation revealed astigmatism and hypermetropia, and he began wearing glasses at 1.5 years of age. Delayed requisition of developmental milestones was evident, as he began walking at 22 months, and said his first words at 26 months. Upon evaluation (30 months), he had severe GDD, with an overall developmental quotient (DQ) of 50. Seizures were not documented. Upon physical examination, weight was 12.1 kg (30th percentile), height was 83.4 cm (10th percentile) and head circumference was 52 cm (95th percentile). Broad forehead, conical fingers, hypotonia and joint hypermobility were noted.

Proband C was evaluated at 35 years of age due to ID, epilepsy and overgrowth. The fourth of five children born to non-consanguineous healthy parents of Arab-Muslim descent, he was born after an uneventful pregnancy and delivery at term (birth weight, 4,500 g). Delay in acquisition of developmental milestones was observed: He walked at the age of 3 years, spoke first words at the age of 4 years. At the age of 8 years he experienced his first generalized seizure, followed by a second and last seizure, with no additional events under anticonvulsant therapy. His growth parameters were reported as above the average, however starting at the age of 10 years he began to grow at an accelerated rate. Upon physical examination at 35 years, morbid obesity, occipital protrusion and upsweeping of frontal hair were noticed, as well as large hands with long and lean fingers. Height was 205 cm (>97th percentile), weight was 150 kg (>97th percentile) and head circumference was 62 cm (>97th percentile). Brain CT was considered normal. Brain MRI demonstrated a 6 x 8 mm non-functioning pituitary lesion which was considered to be an incidental finding without clinical relevance. Hormonal profiling showed decreased testosterone levels.

For all three patients, chromosomal microarray analysis (CMA) and maternal molecular analysis for Fragile X (FRAX) syndrome were normal. For proband B, targeted Sanger sequencing of PTEN was normal.

### Molecular Analysis—Whole Exome Sequencing

**Figure 1:**
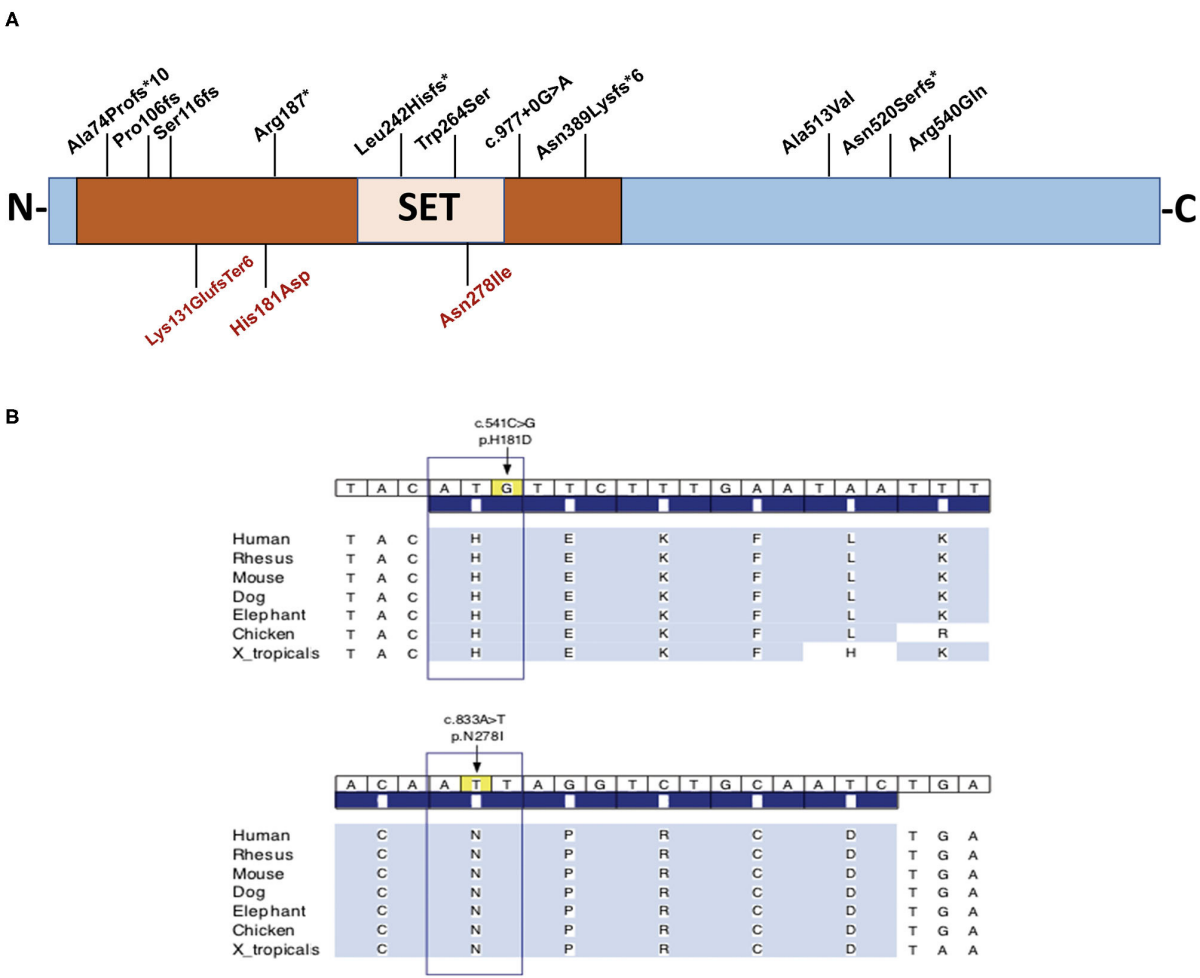
**(A)** Schematic representation of the full length KMT5B protein and the location of disease-causing variants in three unrelated patients, and all previously-reported patients for whom phenotypic features were available (*n* = 14). The variants identified in the three individuals in this report appear on the bottom. **(B)** Conservation of amino acids H181 and N278 throughout evolution. Brown box designates the functional domain of KMT5B as a histone-lysine N-methyltransferase.

In order to reach a molecular diagnosis, trio-WES was pursued for each of the probands. Each of the three probands presented a distinct, *de-novo* heterozygous variant in *KMT5B* (NM_017635.5). Two were of missense type: c.541C > G (p.His181Asp) in proband A, and c.833A > T (p.Asn278Ile) in proband B. In proband C, we identified a c.391_394delAAAG (p.Lys131GlufsTer6) frameshift variant. For probands A and B, harboring the missense variants, the affected amino acid residues are highly conserved throughout evolution (Figure 1), are extremely rare (not found in public databases, including gnomAD, nor in the Sheba Medical Center in-house database) and predicted as damaging/pathogenic by several software (including PolyPhen-2, MutationTaster, FATHMM and SIFT). With regard to the missense variants, the gene pLI and Z score are 1 and 2.79, respectively. The CADD scores for the p.His181Asp and the p.Asn278Ile missense variants were 28.7 and 31, respectively. The p.His181Asp variant was classified as likely-pathogenic (meeting the ACMG criteria: PM2, PP3, PS2), and the two others as pathogenic (ACMG criteria: PM2, PP3, PS2 for the p.Asn278Ile variant and PVS1, PM2, PS2 for p.Lys131Glufs). The p.Asn278Ile is likely to be deleterious to the enzyme activity, as it is located in the SET domain, close to the S-adenosyl-L-methionine and Zinc binding region of the catalytic domain, which are crucial for its function, and in which two pathogenic variants have been previously reported (Figure 1). Table 1 summarizes the disease-causing variants in previously-reported patients, and those described herein.

**Table 1:** Clinical and molecular characteristics of individuals harboring disease-causing variants in *KMT5B* or deletions encompassing *KMT5B*.

**Patient**	**Sex**	**Age at diagnosis**	**Inheritance**	**Disease-causing variant in *KMT5B***	**Intellectual disability**	**Autism/Autistic traits**	**Attention deficit**	**Seizures**	**Dysmorphic and additional features**	**Head circumference (SD)**	**Report**
**Patients harboring a protein-truncating variant in** ***KMT5B*** **(including splice-site variant) or genomic deletions encompassing** ***KMT5B***											
1	F	NA	*de novo*	c.725del, p.(Leu242Hisfs^*^ 30)	+ Mild-moderate	-	+	Febrile		NA	Stessman et al. (4)
2	M	NA	*de novo*	c.1557_1558del, p.(Asn520Serfs^*^ 33)	+ Mild-moderate	+		NA	Unilateral cryptorchidism	NA	Stessman et al. (4)
3	M	NA	Unknown	c.1166dup, p.(Asn389Lysfs^*^6)	+	+		Febrile in infancy	Unilateral pes equinovarus, bilateral pes plano valgus	NA	Stessman et al. (4)
4	F	13 y	*de novo*	c.219delC, p.(Ala74Profs^*^10)	+ Moderate	Autistic traits			Broad large forehead, epicanthus, posteriorly rotated ears, high palate, finger hypermobility	Macrocephaly (4.43)	Faundes et al. (6)
5	M	19 y	*de novo*	c.559C > T, p.(Arg187^*^)	+ Severe	Autistic traits		+	Prominent forehead, thick ear lobes, broad philtrum, an open mouth appearance, synophrys, hypotonia, triangular facial shape, frontal bossing, proptosis, microtia, inverted nipples	Normal (1.93)	Faundes et al. (6)
6	NA	NA	*de novo*	c.315_319delTCCTT, p.(Pro106fs)	+	NA	NA	-	High palate, smooth philtrum, triangular face, low-set ears, cupped ear, myopia, dental crowding, hypotonia, joint hypermobility, failure to thrive, neutropenia, constipation, lymphadenopathy, short stature, long fingers	NA	Trinh et al. (7)
7	NA	NA	*de novo*	c.347_348delCT, p.(Ser116fs)	+	NA	NA	-	Open mouth, strabismus, hypotonia, short femur, tapering pointed ends of distal finger phalanges	Macrocephaly	Trinh et al. (7)
8	F	NA	*de novo*	c.977 + 1G > A	+	+	+	-		Macrocephaly	Stessman et al. (4) and Iossifov et al. (5)
9 (Proband C)	M	35 y	*de novo*	c.391_394delAAAG, (p.Lys131GlufsTer6)	+Profound	NA	NA	Generalized seizures	Overgrowth (>6 SD), morbid obesity, long lean fingers,high arched palate, gingival hypertrophy, almond shaped eyes	Macrocephaly	Current study
10	M	14 y	*de novo*	399.01 Kb deletion	+ Mild	NA	NA	+	Strabismus, scoliosis, down-slanted palpebral fissures, low-set ears, pointed face	Macrocephaly (2)	Faundes et al. (6)
11	M	16 y	*de novo*	839 Kb deletion	+ Mild-moderate	NA	NA		Long oval face, strabismus, ptosis, prominent eyes, protruded ears, open mouth, thick lips, multiple lentigines, long fingers, hypermobility, cryptorchidism, pectus excavatum, overlapping 2nd and 3rd toes; Type I diabetes mellitus	Normal (1.87)	Faundes et al. (6)
**Patients harboring a protein-altering (missense) variant** ***KMT5B***											
12	NA	NA	Maternal	c.1619G > A, p.(Arg540Gln)	+ (IQ 57)	NA		NA	Bilateral epicanthal folds	NA	Stessman et al. (4)
13	M	NA	*de novo*	c.791G > C, p.(Trp264Ser)	+ Moderate	+		–	High forehead, horizontal palpebral fissures, sparse lateral eyebrows, small slightly low ears, wide nasal bridge, wide nasal base, low columella, long toes, long feet, notable fetal finger pads; Combined variable immune deficiency	NA	Stessman et al. (4) and Iossifov et al. (5)
14	M	NA	*de novo*	c.1538C > T, p.(Ala513Val)	+	+	+	Febrile		NA	Stessman et al. (4) and Iossifov et al. (5)
15 (Proband A)	M	12 m	*de novo*	c.541C > G, p.(His181Asp)	+ Profound	NA	NA	–	Broad forehead, strabismus, short neck, mild hypertelorism	Relative macrocephaly	Current study^*^
16 (Proband B)	M	30 m	*de novo*	c.764A>T, p.(Asn255Ile)	+ Profound (DQ 50)	NA	NA	–	Broad forehead, astigmatism, hypermetropia, lactose intolerance, hypotonia, conical fingers, joint hypermobility	Relative macrocephaly	Current study^*^

*DQ, development quotient; IQ, intelligence quotient; NA, not available; SD, standard deviation*.

* *This patient was briefly mentioned in our recent publication: Pode-Shakked et al. (11)*.

### Residue H181 Forms Two Hydrogen Bonds and Residue N278 Forms Five Hydrogen Bonds in the Wild-Type *KMT5B* Structure

**Figure 2:**
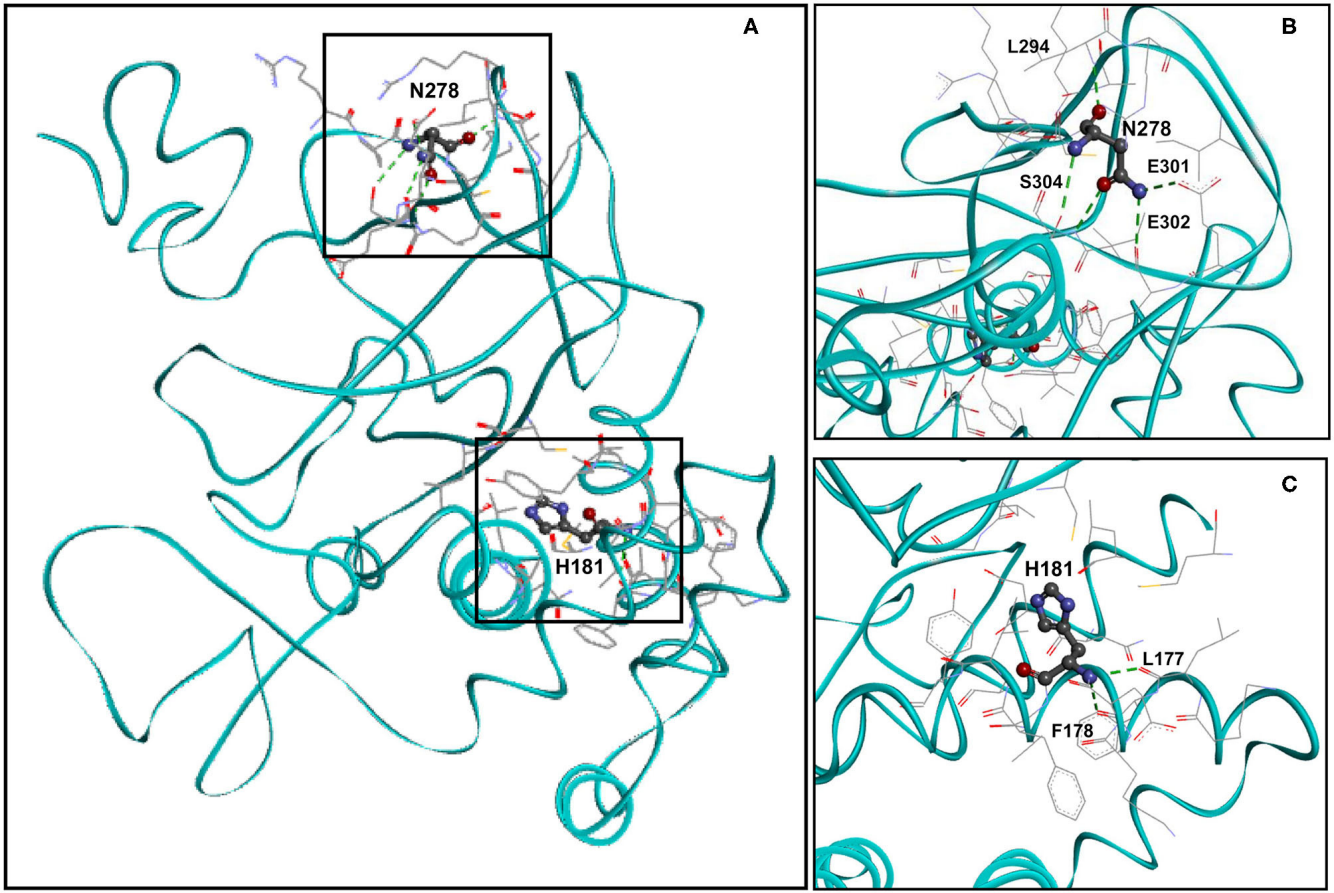
Molecular model of wild-type *KMT5B* **(A)**. *KMT5B* is in cyan. Residues H181 and N278 are shown ball and stick by charge. Residues that are within 5Å distance from H181 or from N278 are shown in stick. Hydrogen bonds are presented in dashed green. **(B)** Close-up of wild-type residue N278 with residues that are within 5Å distance and the residues with which hydrogen bonds are formed. Five hydrogen bonds, presented in dashed green, are formed between N279 and residues L294, E301, E302 and S304. Two hydrogen bonds are formed between N278 and S304. The set of residues (by element, stick) that are within 5Å from N278 are also shown, including: R276, P277, C279, K280, K292, A293, L294, R295, I297, E301, E302, and S304. **(C)** Close-up of wild-type residue H181 with residues that are within 5Å distance and the residues with which hydrogen bonds are formed. Two hydrogen bonds, presented in dashed green, are formed between H181 and residues L177 and F178. The set of residues (by element, stick) that are within 5Å from H181 are also shown, including: K176, L177, F178, K179, E180, V182, F183, I184, Y185, C230, L265, and G266.

We analyzed the spatial location of residues H181 and N278 (identified missense variant sites) and the hydrogen bonds they form. Figure 2A shows the spatial location of residues H181 and N278 (ball and stick), the residues with they form hydrogen bonds (stick) as well as the residues that are with 5Å distance (stick) within wild-type *KMT5B* (cyan, solid ribbon). The hydrogen bonds formed by N278 and H181 in wild-type *KMT5B* are presented in Figures 2B,C, respectively.

### An Additional Hydrogen Bond Is Formed Upon p.H181D Variant

**Figure 3:**
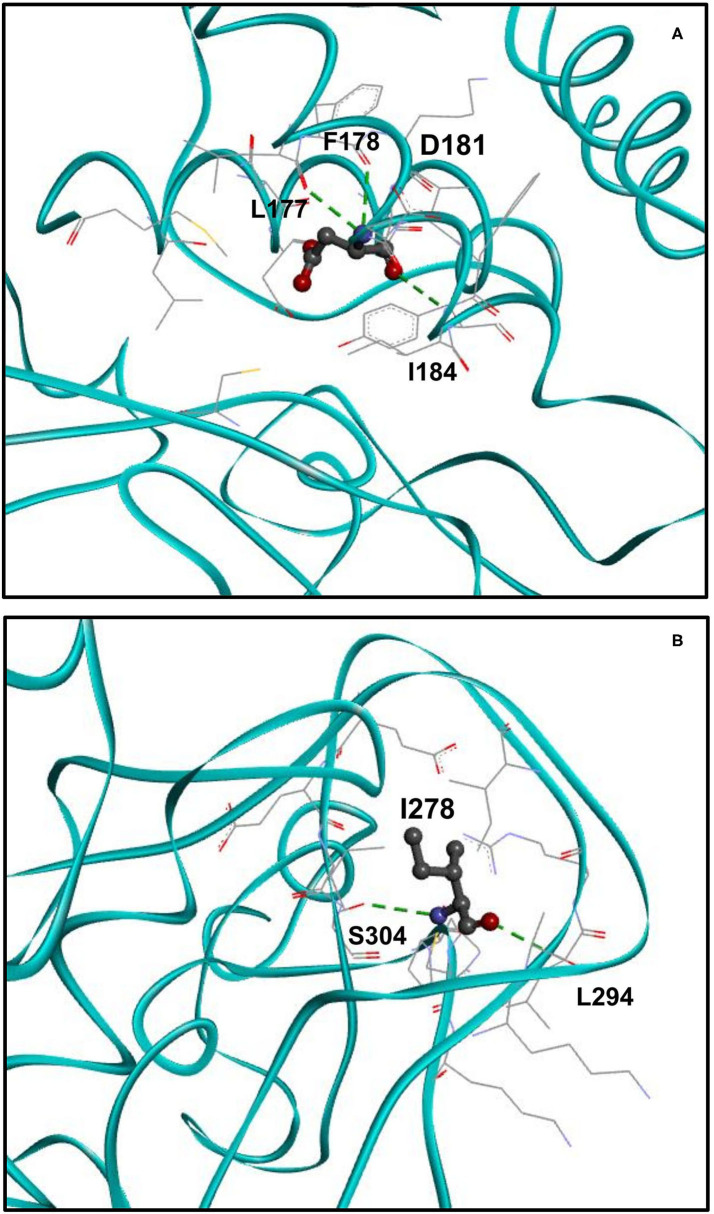
Structure of H181D and N278I mutants. **(A)** Close up view on the structure of H181D mutant. *KMT5B* is in cyan. Residue D181 is shown in ball and stick by charge and the residues with which hydrogen bonds are formed, shown in dashed green, are presented in stick. According to the model, hydrogen bonds are formed between D181 and L177, F178 and I184. **(B)** Close up view on the structure of N278I mutant. Residue I278 is shown in ball and stick by charge and the residues with which hydrogen bonds are formed, shown in dashed green, are presented in stick. Two hydrogen bonds are formed between I278 and S304 and a single hydrogen bond is formed with L294.

We analyzed the spatial location of the identified H181D (p.His181Asp) variant and the hydrogen bonds it forms (Figure 3A). According to the structural model, while WT H181 forms two hydrogen bonds with residues L177 and F178, the mutated H181D model forms an additional bond with I184. In addition, three of the residues that were within 5Å distance from H181 (L176, L265 and G266) are no longer found in such proximity in the H181D mutant structure.

### Three Hydrogen Bonds Are Lost Upon p.N278I Variant

Finally, we analyzed the spatial location of the identified N278I (p.Asn278Ile) variant and its hydrogen bonds (Figure 3B). While WT N278 forms five hydrogen bonds with residues L294, E301, E302 and two hydrogen bonds with S304, the mutated N278I model forms only two hydrogen bonds. That is, one of the hydrogen bonds with S304 is lost and the hydrogen bonds with E301 and E302 are lost as well. In addition, I303 that was not within 5Å distance from N278 is found to be within the 5Å distance in the N278I mutant structure.

## Discussion

The association of disease-causing variants in *KMT5B* with neurodevelopmental disorders, with or without ASD and/or seizures, has only recently been recognized (4, 6, 7). In this study, we report three individuals with *de novo* variants in this gene, characterized also by macrocephaly and ophthalmological involvement.

The enzyme *KMT5B*, as well as its highly homologous counterpart KMT2C, is a lysine methyltransferase known to mainly affect the K20 site on histone protein H4 (H4K20) (13, 14). There is accumulating evidence establishing the involvement of H4K20 methylation in brain development (9).

The majority of the previously-reported variants in *KMT5B* are protein-truncating variants (PTVs) causing loss of function. These include 6 indel variants, one nonsense variant and a splice-site variant. Two additional patients harbor a genomic deletion encompassing *KMT5B*. Given these variants found in affected individuals, the pLI scores and the aforementioned murine models (8), the mechanism of disease for this subgroup of patients is haploinsufficiency. In contrast, three previously-reported patients have protein-altering (missense) variants (PAVs). These might result in an alternate mechanism, such as gain-of-function or a dominant-negative effect, however this remains to be elucidated. As presented in Table 1, there are no striking differences between patients with PTVs vs. PAVs with regard to the degree of intellectual disability or the presence of other phenotypic features (autism, seizures, macrocephaly, overgrowth, etc.). Therefore, no clear genotype-phenotype correlations can be safely deduced based on these small numbers of patients.

Using computational 3D modeling of the *KMT5B* protein, we demonstrate the predicted effect of the two missense variants on hydrogen bonds with nearby residues and on the overall 3D protein structure, further underscoring their pathogenicity.

About 50 histone lysine methyltransferases are recognized in humans. Most of them contain a SET domain as their catalytic domain (15, 16), and along with the post-SET domains are involved in substrate and cofactor binding (17). A multiple sequence alignment of SET domains from different human histone methyltransferases (18) has shown that residue N278, which is found within the SET domain, is evolutionarily conserved and is located in the vicinity of C275 which binds the Zn ion (18).

Hydrogen bonds are one of the major protein structure determinants and their making and breaking profoundly affects the activity of proteins (19, 20). Previous studies have shown that mutation of F281 to A, as well as other mutations along the SET domain, abolishes histone methyltransferase activity (18). Considering the proximity of N278 to F281 and the loss of three hydrogen bonds upon mutation to I, impaired stability may be implied affecting histone methyltransferase activity. As for the H181D mutation, some predictions (e.g. by InterPro and Pfam) suggest it might be considered part of the SET domain as well, so its effect may also be impaired catalytic activity.

While the neurodevelopmental phenotype associated with *KMT5B* is variable and has yet to be fully characterized, several of its components are becoming evident. Developmental delay and/or ID of varying severity seems to be universal, and was noted in 16/16 (100%) of the patients for whom there is clinical data available in the literature, including the three reported herein. Additional features reported in some individuals include ASD or autistic traits (7/16, 44%; 5/9 of patients with PTVs, 2/5 with PAVs), hypotonia, and seizures (including febrile seizures) (6/16, 37.5%; 4/9 of PTVs, 1/5 with PAVs). Macrocephaly (or relative macrocephaly) is another characteristic (albeit not universal: 7/16, 44%). Brain MRI findings have been noted occasionally, including corpus callosum hypoplasia, hydrocephalus and cerebral ventricular enlargement. Dysmorphic facial features have been described, however these did not seem to be consistent or lead to a recognizable facial phenotype. Nevertheless, several features did recur among some affected individuals, such as ophthalmological involvement (strabismus in 5/16, 31%, ptosis), broad forehead (5/16, 31%), epicanthal folds, high palate, joint hypermobility and long or conical fingers. Finally, overgrowth of varied extent was noted in 5/16 (31%) of patients.

The differential diagnosis of GDD/ID accompanied by macrocephaly is diverse and includes numerous inherited disorders, such as PTEN hamartoma syndromes, Fragile X and Canavan disease, among others (21). Interestingly, macrocephaly (or relative macrocephaly) has been previously described as a common feature of additional neurodevelopmental disorders associated with variants in other genes of the lysine methyltransferase family, such as *KMT2E* (22). However, the pathomechanistic association between this group of proteins and abnormal brain size has yet to be elucidated. While macrocephaly and/or overgrowth were not consistently described among patients with *KMT5B* variants, we suggest that this genetic diagnosis should be entertained in undiagnosed patients with ID and macrocephaly or overgrowth.

Finally, due to the small number of patients with point mutations in *KMT5B* reported to date, and the limited phenotypic information available for most of them, it is still early to determine whether or not genotype-phenotype correlations exist for this entity, and whether there are recognizable differences in terms of disease severity between patients harboring loss-of-function vs. missense variants. Additionally, while most reported patients were male, the overall small number of patients precludes us from drawing conclusions as to possible sex effects on the phenotype. This is particularly interesting given the aforementioned differential effects by sex suggested by behavioral murine studies (8). Future studies detailing clinical and molecular characteristics of larger cohorts of patients with *KMT5B* variants will shed light on these unanswered questions.

To conclude, our findings expand the current knowledge regarding the phenotype and variant spectrum of *KMT5B*-associated ID, and suggest that this entity should be considered in the differential diagnosis of ID accompanied by relative macrocephaly and/or overgrowth.

## Data Availability Statement

The data sets presented in this article are not readily available, since parental consent was not obtained for sharing the raw data with public databases. Requests to access the databases should be directed to the corresponding author.

## Ethics Statement

The studies involving human participants were reviewed and approved by Institutional Review Boards (IRBs) of the Sheba Medical Center and Beilinson Medical Center. Written informed consent to participate in this study was provided by the participant's legal guardian/next of kin.

## Author Contributions

AE, LG, and BP-S initiated the study, recruited patients, analyzed the results, wrote the manuscript, and critically reviewed it. GZ, YG, AR-R, AS, OS-C, NP-S, NR-S, EP, MF, and MS obtained clinical data and analyzed it and critically reviewed the manuscript. OB, IB-J, EJ, and LB interpreted the exome sequencing data. VK provided the 3D protein structures and their analyses. All authors critically reviewed the manuscript.

## Conflict of Interest

VK was employed by Bioinformatics Consulting. The remaining authors declare that the research was conducted in the absence of any commercial or financial relationships that could be construed as a potential conflict of interest.

## Publisher's Note

All claims expressed in this article are solely those of the authors and do not necessarily represent those of their affiliated organizations, or those of the publisher, the editors and the reviewers. Any product that may be evaluated in this article, or claim that may be made by its manufacturer, is not guaranteed or endorsed by the publisher.
