# Supplementary material for: Refining the Phenotypic Spectrum of KMT5B-Associated Developmental Delay
Source: Front Pediatr. 2022 Mar 30;10:844845. doi: 10.3389/fped.2022.844845 (PMC9005902; doi:10.3389/fped.2022.844845)
Supplement: Supplementary file 1 [file Data_Sheet_1.docx]

**Supplementary data**

*Whole Exome Sequencing* (Detailed methods)

For probands A and B and their parents, Whole Exome Sequencing (WES) was performed on genomic DNA at the Bioinformatics Unit, Sheba Medical Center, using the Twist Human Core Exome and the Sure Select Human All Exon V6, and Illumina 2500 sequencing technology. For each sample, paired end reads (2 × 100bp for Twist and 2 × 150 bp for Sure Select) were obtained, processed and mapped to the genome. We used the BWA MEM algorithm (version 0.7.15)^1^ for alignment of the sequence reads to the human reference genome (hg19). The HaplotypeCaller algorithm of GATK version 3.8 was applied for variant calling, as recommended in the best practice pipeline^2^. KGG-seq v.1.0 was used for annotation of identified variants^3^, and in-house scripts were applied for filtering based on family pedigree and local dataset of variants detected in previous sequencing projects (in-house cohort of 2501 samples) and the gnomAD dataset^4^. Pathogenicity of the variants was also evaluated according to American College of Medical Genetics and Genomics (ACMG) criteria^5^, using VarSome^6^.

For proband C and his parents, WES was performed at the Raphael Recanati Genetics Institute. The family samples were sequenced as part of the clinical service by the CeGat laboratory (CeGaT GmbH, Tuebingen, Germany). Targeted capture of protein-coding regions was performed using the Twist Human Core Exome Plus Kit (Twist Bioscience, San Francisco, CA, USA). Paired-end libraries were prepared from captured fragments and sequenced on the Illumina NovaSeq 6000 platform (Illumina, San Diego, CA, USA). Sequencing was performed to achieve at least 97 percent of target bases covered at 20x or greater (95% at >100x). The FASTQ files, along with information on phenotypes using human phenotype ontology (HPO) terms and family structure, were uploaded into Emedgene’s HIPAA-compliant platform (Emedgene Technologies, Ltd, Mazor, Israel) and analyzed as described previously^7^. The parameters used for variant interpretation included moderate and high variant quality (including mapping quality ≥ 45 and depth ≥ 10), population frequency (1% or 5% for dominant or recessive inheritance, respectively) and variant severity; excluding variants in 5'/3' UTR, non-coding transcript exon variant, intron variant, non-coding transcript variant, and upstream/downstream gene variants. In addition, auto-analysis identified approximately ten variants highly likely to solve the case. Analysis of copy number variants was not performed.

References

1. Li, H. & Durbin, R. (2009). Fast and accurate short read alignment with Burrows-Wheeler transform. Bioinformatics 25, 1754-60.
2. McKenna, A., Hanna, M., Banks, E., Sivachenko, A., Cibulskis, K., Kernytsky, A., *et al*. (2010). The genome analysis toolkit: a MapReduce framework for analyzing next-generation DNA sequencing data. Genome Res. 20, 1297–1303.
3. Li, M.X., Gui, H.S., Kwan, J.S., Bao, S.Y., Sham, P.C. (2012). A comprehensive framework for prioritizing variants in exome sequencing studies of Mendelian diseases. Nucleic Acids. Res. 40, e53.
4. Karczewski, K.J., Francioli, L.C., Tiao, G., Cummings, B.B., [Alföldi](https://pubmed.ncbi.nlm.nih.gov/?sort=date&term=Alf%C3%B6ldi+J&cauthor_id=32461654), J., Wang, Q., *et al*. (2020). The mutational constraint spectrum quantified from variation in 141,456 humans. Nature 581(7809), 434-443.
5. Richards, S., Aziz, N., Bale, S., Bick, D., Das, S., Gastier-Foster, J., *et al*. (2015). Standards and guidelines for the interpretation of sequence variants: a joint consensus recommendation of the American College of Medical Genetics and Genomics and the Association for Molecular Pathology. Genet. Med. 17(5), 405-24.
6. Kopanos, C., Tsiolkas, V., Kouris, A., Chapple, C.E., Albarca Aguilera, M., Meyer, R., *et al*. (2019). VarSome: the human genomic variant search engine. Bioinformatics 35(11), 1978-1980.
7. Basel-Salmon, L., Orenstein, N., Markus-Bustani, K., Ruhrman-Shahar, N., Kilim, Y., Magal, N., *et al*. (2019). Improved diagnostics by exome sequencing following raw data reevaluation by clinical geneticists involved in the medical care of the individuals tested. Genet. Med. 21(6), 1443-1451.
